# 1,8-Di­aza­bicyclo­[5.4.0]undec-7-en-8-ium bromido­(phthalocyaninato)zincate

**DOI:** 10.1107/S160053681401157X

**Published:** 2014-05-24

**Authors:** Bartosz Przybył, Jan Janczak

**Affiliations:** a Institute of Low Temperature and Structural Research, Polish Academy of Sciences, Okólna 2, Wrocław, 50-422, Poland

## Abstract

The title compound, (C_9_H_17_N_2_)[ZnBr(C_32_H_16_N_8_)], contains a bromido­(phthalocyaninato)zincate anion and a protonated 1,8-di­aza­bicyclo­[5.4.0]undece-7-ene cation, [DBUH]^+^. The central Zn^II^ atom has a distorted square-pyramidal geometry, with four iso­indole N atoms of the macrocycle in equatorial positions and a bromide ion in the axial position. The latter has a relatively high displacement parameter, but no evidence for disorder was obtained. The central Zn^II^ atom is displaced by 0.488 (3) Å from the mean plane defined by the four iso­indole N atoms. The [DBUH]^+^ cation is involved in an almost linear N—H⋯Br hydrogen bond. In the crystal, π–π inter­actions lead to a relatively short distance of 3.366 (3) Å between the phthalocyaninate rings.

## Related literature   

For background information on phthalocyanines, see: Nyokong *et al.* (1987 ▶); Gregory (2000 ▶); Leznoff & Lever (1996 ▶); Tedesco *et al.* (2003 ▶); Ormond & Freeman (2013 ▶). For related structures, see: Kobayashi *et al.* (1971 ▶); Mossoyan-Deneux *et al.* (1985 ▶); Zeng *et al.* (2005 ▶); Del Sole *et al.* (2005 ▶); Kubiak *et al.* (2007 ▶); Yang *et al.* (2008 ▶); Janczak *et al.* (2009 ▶, 2011 ▶); Janczak & Kubiak (2009 ▶); Li *et al.* (2011 ▶); Przybył & Janczak (2014 ▶).
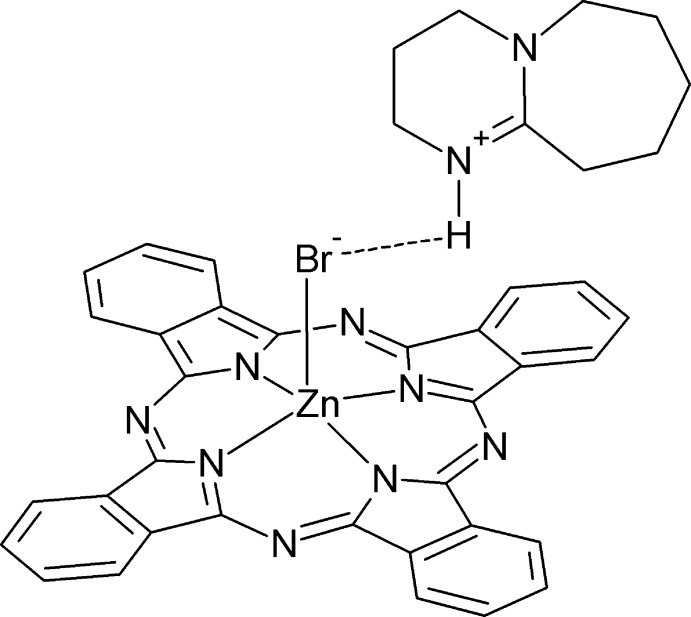


## Experimental   

### 

#### Crystal data  

(C_9_H_17_N_2_)[ZnBr(C_32_H_16_N_8_)]
*M* *_r_* = 811.05
Monoclinic, 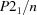

*a* = 12.4336 (8) Å
*b* = 22.9278 (16) Å
*c* = 13.3267 (9) Å
β = 113.266 (4)°
*V* = 3490.2 (4) Å^3^
*Z* = 4
Mo *K*α radiation
μ = 1.90 mm^−1^
*T* = 295 K
0.35 × 0.21 × 0.19 mm

#### Data collection  

Kuma KM-4 with CCD detector diffractometer
Absorption correction: numerical (*CrysAlis RED*; Oxford Diffraction, 2008 ▶) *T*_min_ = 0.562, *T*_max_ = 0.725
42041 measured reflections
8492 independent reflections
4908 reflections with *I* > 2σ(*I*)
*R*_int_ = 0.045

#### Refinement  

*R*[*F* ^2^ > 2σ(*F* ^2^)] = 0.057
*wR*(*F* ^2^) = 0.143
*S* = 1.00
8492 reflections
478 parameters
H atoms treated by a mixture of independent and constrained refinement
Δρ_max_ = 0.76 e Å^−3^
Δρ_min_ = −1.26 e Å^−3^

### 

Data collection: *CrysAlis CCD* (Oxford Diffraction, 2008 ▶); cell refinement: *CrysAlis RED* (Oxford Diffraction, 2008 ▶); data reduction: *CrysAlis RED*; program(s) used to solve structure: *SHELXS97* (Sheldrick, 2008 ▶); program(s) used to refine structure: *SHELXL97* (Sheldrick, 2008 ▶); molecular graphics: *DIAMOND* (Brandenburg & Putz, 2006 ▶); software used to prepare material for publication: *SHELXL97*.

## Supplementary Material

Crystal structure: contains datablock(s) I. DOI: 10.1107/S160053681401157X/fj2673sup1.cif

Structure factors: contains datablock(s) I. DOI: 10.1107/S160053681401157X/fj2673Isup2.hkl

CCDC reference: 1003951

Additional supporting information:  crystallographic information; 3D view; checkCIF report

## Figures and Tables

**Table 1:** Hydrogen-bond geometry (Å, °)

*D*—H⋯*A*	*D*—H	H⋯*A*	*D*⋯*A*	*D*—H⋯*A*
N10—H10⋯Br1	0.87 (2)	2.44 (2)	3.281 (4)	163 (2)

**Fig. 1.**
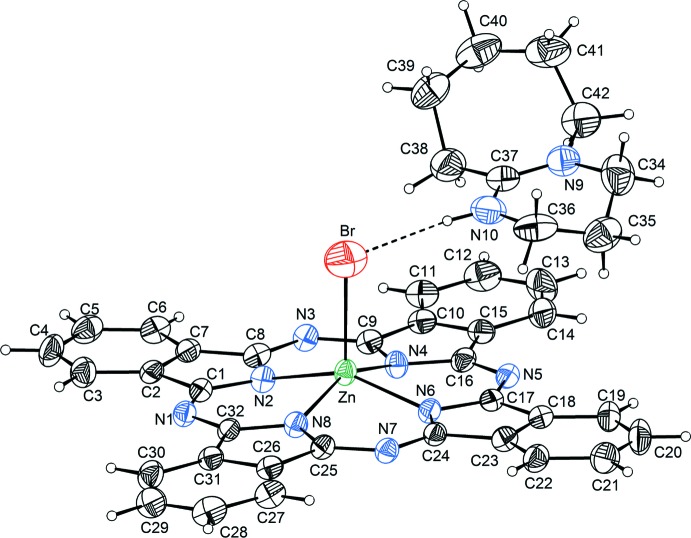
Asymmetric part of the unit cell of 1, showing the atom-numbering scheme. Displacement ellipsoids are drawn at the 50% probability level.

**Fig. 2.**
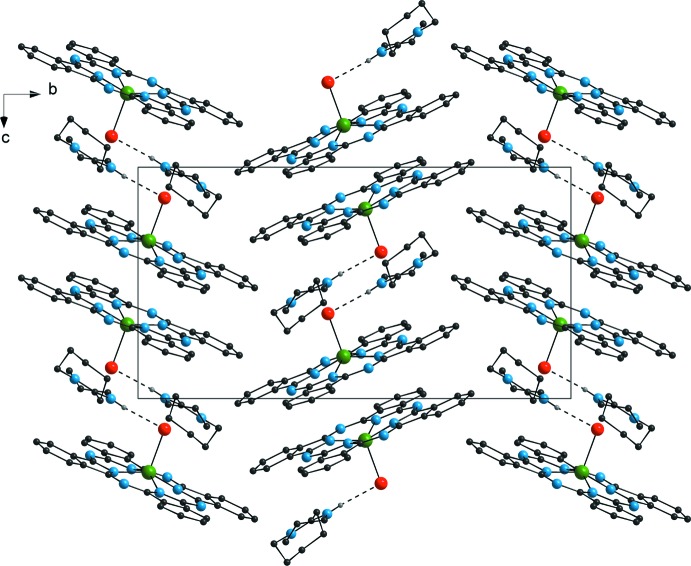
Unit cell packing in 1, viewed along a. All atoms are represented by spheres. H atoms, except one involved in hydrogen-bond formation, have been omitted for clarity.

## supplementary crystallographic information

### 1. Comment

Phthalocyanines and metallophthalocyanines (Pcs and MPcs) arouses inter­est because of exhibition of many important features which gives potential application in many fields from industry to medicine (Leznoff & Lever, 1996; Gregory, 2000). The utility of MPc complexes is limited by their relatively low solubility in the most organic solvents. The solubility of MPcs can be enhanced by substitution of the H atoms of Pc macrocycle or/and by ligation an additional ligand the the metal centre of MPcs. Both ways of modification of MPc's lead to improvment of their solubility, due to decreasing of the π···π inter­actions between the Pc macrocycles as well as lowering their aggregation in solution. ZnPc and its derivatives, due to strong absorption in the red region of visible radiation and high triplet quantum yield and long lifetimes, are intensively studied as potential agents in photodynamic therapy (Tedesco *et al.*, 2003; Ormond & Freeman, 2013).

Complex 1 crystallizes as ionic compound in the centrosymmetric space group of monoclinic system. The blue-violet crystals of 1 are built up from bromido(phthalocyaninato)zinc(II) anion (ZnPcBr^-^) and 1,8-di­aza­bicyclo­[5.4.0]undec-7-en-8-ium cation (DBUH^+^) (Fig. 1). Both oppositely charged units inter­act via N—H10···Br hydrogen-bond, in which DBUH^+^ plays role of a donor (Table 1). Due to presence of DBU that accepts H^+^, phthalocyaninato macrocyle maintains -2 oxidation state, therefore the complex 1 is diamagnetic as show the magnetic susceptibility measurements. This is in contrast to the chloro­(phthalocyaninato)zinc(II) in which ZnPc is oxidised and has +1 charge, and the metal centre is coordinated by phthalocyaninato anion with π-radical character, Pc^-.^ (Mossoyan-Deneux *et al.*, 1985). As an effect of monaxial Zn—Br bond formation, Zn(II) atom exhibits displacement from a plane defined by four iso­indole nitro­gen atoms of Pc(2-) (N_4_ plane) by 0.488 (3) Å. This is a value situated in the typical range in comparison with other 4+1 complexes of ZnPc reported in the literature (Table S1). Additionally, pyramidal coordination environment of Zn(II) centre is slightly distorted, angle between the normal to the N_4_ plane and the Zn—Br bond is equal 1.65 (5) ° that is inclined towards the hydrogen-bond. In reported structure Pc(2-) macrocycle has nearly flat conformation though deformation from flat to saucer-like shape is very common among 4+1 MPc complexes arranged in the back-to-back fashion in solids. Almost all nitro­gen and carbon atoms of Pc(2-) exhibit displacement from the mean plane of these atoms smaller than 0.1 Å (only three peripheral carbon atoms slightly exceed this value). Complex of ZnPc with chloride (Mossoyan-Deneux *et al.*, 1985) also exhibits nearly flat conformation of Pc(2-) macrocycle, thus deformation of the molecule into saucer-like shape is probably caused by inter­action with bulky ligands like amines or pyridine derivatives (Table S1). Mutual arrangement of the complex molecules exhibits typical back-to-back fashion, with the distance 3.366 (3) Å, as a consequence of π···π inter­action between Pc(2-) macrocycles. Back-to-back oriented molecules exhibits relatively small shift in plane of Pc(2-) macrorings (Fig. 2).

Thanks to DBU molecule that plays role of acceptor H^+^, ligation of ZnPc by Br^-^ with the formation of the ionic complex (ZnPcBr^-^)(DBUH^+^) maintains -2 oxidation state of the Pc macrocycle . Thus ligation of the central Zn atom of ZnPc by Br^-^ in 1 does not change the colouring properties compared with the parent ZnPc pigment since the energy gap of HOMO-LUMO level is not disturbed. The Q band assigned to HOMO-LUMO transition is observed at almost the same wavelength of ~673 nm in both complexes (ZnPcBr^-^)(DBUH^+^) and ZnPc (Nyokong *et al.*, 1987). However, the ionic compound 1 is significantly better soluble than ZnPc pigment and therefore this feature of 1 widnes its potential utility.

### 2. Experimental

Crystals of 1 were obtained by heating of complex ZnPc—DBU, well characterized in literature (Del Sole *et al.* 2005, Janczak *et al.*, 2011), with *n*-pentano­thiol slightly acidified with HBr under solvothermal conditions (evacuated glass elongated ampoule, 130 °C, 5 days). Elemental analysis of the ionic (ZnPcBr^-^)(DBUH^+^) crystals was performed with energy dispersive spectroscopy (EDS) as well as with a Perkin-Elmer 2400 elemental analyser. EDS spectra were acquired and analysed using an EDAX Pegasus XM4 spectrometer with SDD Apollo 4D detector mounted on a FEI Nova NanoSEM 230 microscope. Found: Zn, 8.00; Br, 9.95; C, 60.45; N, 17.17 and H, 4.20%. Calculated for C_41_H_33_N_10_ZnBr: Zn, 8.06; Br, 9.84; C, 60.72; N, 17.27 and H, 4.10%.

The temperature dependence of the magnetic susceptibility was performed on Quantum Design SQUID magnetometer (San Diego, CA). The data were recorded at a magnetic field of 0.5 T between 1.8 and 300 K on the sample of 50 mg. Electronic absorption spectrum in solution (CARY Varian SE UV-Vis-NIR spectrometer) exhibits typical for Pc macrocycle two bands: Q (~673 nm, logε=5.55) and B (~350 nm,logε=4.22).

### 3. Refinement

Crystal data, data collection and structure refinement details are summarized in Table 1. During refinement of the crystal structure we have seen the relatively high thermal parameters of Br coordinated to ZnPc. Therefore, the composition of the crystal has been checked with energy dispersive spectroscopy (EDS) as well as with a Perkin-Elmer 2400 elemental analyser. Elemental analysis evidently points to 1:1 proportion of Zn:Br. Therefore the refinement of the crystal structure was performed with the occupation factor of 1 for Br. Hydrogen atoms were placed in calculated positions and refined using a riding model with C—H = 0.97 Å and *U*_iso_(H) = 1.5*U*_eq_(C) for aliphatic and C—H = 0.93 Å and *U*_iso_(H) = 1.2*U*_eq_(C) for aromatic moieties. H atom involved in N—H···Br bond was localized in difference maps and refined with N—H distance restrain of 0.87 (2) Å.

### Crystal data

**Table:**

(C_9_H_17_N_2_)[ZnBr(C_32_H_16_N_8_)]	*F*(000) = 1656
*M**_r_* = 811.05	*D*_x_ = 1.544 Mg m^−^^3^*D*_m_ = 1.54 Mg m^−^^3^*D*_m_ measured by floatation
Monoclinic, *P*2_1_/*n*	Mo *K*α radiation, λ = 0.71073 Å
Hall symbol: -P 2yn	Cell parameters from 3452 reflections
*a* = 12.4336 (8) Å	θ = 2.6–27.5°
*b* = 22.9278 (16) Å	µ = 1.90 mm^−^^1^
*c* = 13.3267 (9) Å	*T* = 295 K
β = 113.266 (4)°	Parallelepiped, blue-violet
*V* = 3490.2 (4) Å^3^	0.35 × 0.21 × 0.19 mm
*Z* = 4	

### Data collection

**Table:**

Kuma KM-4 with CCD detector diffractometer	8492 independent reflections
Radiation source: fine-focus sealed tube	4908 reflections with *I* > 2σ(*I*)
Graphite monochromator	*R*_int_ = 0.045
ω scan	θ_max_ = 28.8°, θ_min_ = 2.6°
Absorption correction: numerical (*CrysAlis RED*; Oxford Diffraction, 2008)	*h* = −16→12
*T*_min_ = 0.562, *T*_max_ = 0.725	*k* = −30→30
42041 measured reflections	*l* = −17→17

### Refinement

**Table:**

Refinement on *F*^2^	Primary atom site location: structure-invariant direct methods
Least-squares matrix: full	Secondary atom site location: difference Fourier map
*R*[*F*^2^ > 2σ(*F*^2^)] = 0.057	Hydrogen site location: inferred from neighbouring sites
*wR*(*F*^2^) = 0.143	H atoms treated by a mixture of independent and constrained refinement
*S* = 1.00	*w* = 1/[σ^2^(*F*_o_^2^) + (0.076*P*)^2^] where *P* = (*F*_o_^2^ + 2*F*_c_^2^)/3
8492 reflections	(Δ/σ)_max_ < 0.001
478 parameters	Δρ_max_ = 0.76 e Å^−^^3^
0 restraints	Δρ_min_ = −1.26 e Å^−^^3^

### Special details

**Table:**

Geometry. All e.s.d.'s (except the e.s.d. in the dihedral angle between two l.s. planes) are estimated using the full covariance matrix. The cell e.s.d.'s are taken into account individually in the estimation of e.s.d.'s in distances, angles and torsion angles; correlations between e.s.d.'s in cell parameters are only used when they are defined by crystal symmetry. An approximate (isotropic) treatment of cell e.s.d.'s is used for estimating e.s.d.'s involving l.s. planes.
Refinement. Refinement of *F*^2^ against ALL reflections. The weighted *R*-factor *wR* and goodness of fit *S* are based on *F*^2^, conventional *R*-factors *R* are based on *F*, with *F* set to zero for negative *F*^2^. The threshold expression of *F*^2^ > σ(*F*^2^) is used only for calculating *R*-factors(gt) *etc*. and is not relevant to the choice of reflections for refinement. *R*-factors based on *F*^2^ are statistically about twice as large as those based on *F*, and *R*- factors based on ALL data will be even larger.

### Fractional atomic coordinates and isotropic or equivalent isotropic displacement parameters (Å^2^)

**Table:**

	*x*	*y*	*z*	*U*_iso_*/*U*_eq_	
Zn1	0.45113 (3)	0.025014 (16)	0.31931 (3)	0.02443 (12)	
Br1	0.34125 (5)	0.06134 (3)	0.13296 (5)	0.07214 (19)	
N1	0.4406 (2)	0.14541 (12)	0.4657 (2)	0.0260 (6)	
N2	0.3569 (2)	0.04946 (12)	0.4069 (2)	0.0262 (6)	
N3	0.2243 (2)	−0.03246 (12)	0.3688 (2)	0.0266 (6)	
N4	0.3855 (2)	−0.05667 (12)	0.3176 (2)	0.0257 (6)	
N5	0.5072 (2)	−0.11056 (12)	0.2462 (2)	0.0274 (7)	
N6	0.5907 (2)	−0.01490 (11)	0.3055 (2)	0.0241 (6)	
N7	0.7220 (2)	0.06751 (12)	0.3406 (2)	0.0255 (6)	
N8	0.5615 (2)	0.09175 (12)	0.3933 (2)	0.0255 (6)	
C1	0.3601 (3)	0.10425 (14)	0.4491 (3)	0.0241 (7)	
C2	0.2578 (3)	0.11214 (15)	0.4761 (3)	0.0254 (7)	
C3	0.2179 (3)	0.15852 (16)	0.5191 (3)	0.0360 (9)	
H3	0.2594	0.1934	0.5376	0.043*	
C4	0.1140 (3)	0.15119 (18)	0.5335 (3)	0.0392 (10)	
H4	0.0851	0.1817	0.5615	0.047*	
C5	0.0527 (3)	0.09887 (18)	0.5065 (3)	0.0374 (9)	
H5	−0.0170	0.0953	0.5163	0.045*	
C6	0.0927 (3)	0.05206 (16)	0.4655 (3)	0.0310 (8)	
H6	0.0522	0.0169	0.4497	0.037*	
C7	0.1959 (3)	0.05924 (15)	0.4485 (3)	0.0257 (8)	
C8	0.2596 (3)	0.02122 (15)	0.4053 (3)	0.0257 (7)	
C9	0.2820 (3)	−0.06799 (14)	0.3283 (3)	0.0259 (8)	
C10	0.2418 (3)	−0.12622 (15)	0.2871 (3)	0.0282 (8)	
C11	0.1426 (3)	−0.15806 (16)	0.2767 (3)	0.0351 (9)	
H11	0.0886	−0.1438	0.3030	0.042*	
C12	0.1275 (4)	−0.21193 (17)	0.2256 (4)	0.0445 (11)	
H12	0.0614	−0.2339	0.2167	0.053*	
C13	0.2086 (4)	−0.23419 (18)	0.1871 (4)	0.0460 (11)	
H13	0.1959	−0.2707	0.1542	0.055*	
C14	0.3078 (3)	−0.20285 (15)	0.1971 (3)	0.0376 (9)	
H14	0.3614	−0.2172	0.1704	0.045*	
C15	0.3235 (3)	−0.14865 (15)	0.2493 (3)	0.0282 (8)	
C16	0.4146 (3)	−0.10403 (14)	0.2715 (3)	0.0274 (8)	
C17	0.5885 (3)	−0.06958 (14)	0.2633 (3)	0.0260 (8)	
C18	0.6887 (3)	−0.07754 (16)	0.2344 (3)	0.0295 (8)	
C19	0.7285 (3)	−0.12345 (17)	0.1915 (3)	0.0371 (9)	
H19	0.6885	−0.1588	0.1745	0.044*	
C20	0.8312 (4)	−0.11469 (19)	0.1750 (4)	0.0459 (11)	
H20	0.8592	−0.1448	0.1453	0.055*	
C21	0.8930 (3)	−0.06286 (18)	0.2010 (3)	0.0402 (10)	
H21	0.9618	−0.0591	0.1897	0.048*	
C22	0.8530 (3)	−0.01619 (17)	0.2442 (3)	0.0324 (8)	
H22	0.8930	0.0191	0.2604	0.039*	
C23	0.7526 (3)	−0.02415 (15)	0.2619 (3)	0.0285 (8)	
C24	0.6867 (3)	0.01334 (14)	0.3065 (3)	0.0240 (7)	
C25	0.6642 (3)	0.10279 (14)	0.3824 (3)	0.0238 (7)	
C26	0.7048 (3)	0.16120 (14)	0.4240 (3)	0.0259 (8)	
C27	0.8037 (3)	0.19329 (16)	0.4326 (3)	0.0325 (9)	
H27	0.8576	0.1789	0.4064	0.039*	
C28	0.8176 (3)	0.24766 (17)	0.4819 (3)	0.0388 (10)	
H28	0.8823	0.2702	0.4885	0.047*	
C29	0.7384 (3)	0.26927 (16)	0.5212 (3)	0.0386 (9)	
H29	0.7520	0.3054	0.5556	0.046*	
C30	0.6399 (3)	0.23853 (15)	0.5107 (3)	0.0343 (9)	
H30	0.5853	0.2538	0.5351	0.041*	
C31	0.6246 (3)	0.18372 (14)	0.4624 (3)	0.0253 (8)	
C32	0.5330 (3)	0.13890 (14)	0.4404 (3)	0.0226 (7)	
N9	0.2108 (3)	−0.14631 (15)	−0.0847 (3)	0.0429 (8)	
C34	0.3084 (4)	−0.1587 (2)	−0.1199 (4)	0.0594 (13)	
H34A	0.3181	−0.2006	−0.1233	0.089*	
H34B	0.2896	−0.1429	−0.1923	0.089*	
C35	0.4196 (4)	−0.1325 (2)	−0.0422 (4)	0.0561 (12)	
H35A	0.4797	−0.1363	−0.0710	0.084*	
H35B	0.4458	−0.1530	0.0271	0.084*	
C36	0.4004 (4)	−0.0697 (2)	−0.0254 (4)	0.0532 (12)	
H36A	0.4691	−0.0541	0.0332	0.080*	
H36B	0.3888	−0.0480	−0.0914	0.080*	
N10	0.3005 (3)	−0.06292 (16)	0.0010 (3)	0.0476 (9)	
H10	0.2977 (9)	−0.0324 (8)	0.0388 (8)	0.057*	
C37	0.2124 (3)	−0.09974 (18)	−0.0279 (3)	0.0395 (10)	
C38	0.1153 (4)	−0.0872 (2)	0.0084 (4)	0.0559 (12)	
H38A	0.1389	−0.0553	0.0602	0.084*	
H38B	0.1036	−0.1212	0.0461	0.084*	
C39	−0.0027 (4)	−0.0713 (2)	−0.0856 (4)	0.0540 (12)	
H39A	−0.0530	−0.0533	−0.0543	0.081*	
H39B	0.0121	−0.0426	−0.1321	0.081*	
C40	−0.0669 (4)	−0.1216 (2)	−0.1551 (4)	0.0629 (14)	
H40A	−0.0897	−0.1479	−0.1100	0.094*	
H40B	−0.1382	−0.1068	−0.2117	0.094*	
C41	−0.0026 (4)	−0.1557 (2)	−0.2082 (4)	0.0668 (15)	
H41A	0.0188	−0.1299	−0.2549	0.100*	
H41B	−0.0548	−0.1852	−0.2545	0.100*	
C42	0.1090 (4)	−0.18605 (19)	−0.1279 (4)	0.0546 (12)	
H42A	0.0936	−0.2018	−0.0673	0.082*	
H42B	0.1281	−0.2184	−0.1648	0.082*	

### Atomic displacement parameters (Å^2^)

**Table:**

	*U*^11^	*U*^22^	*U*^33^	*U*^12^	*U*^13^	*U*^23^
Zn1	0.0227 (2)	0.0204 (2)	0.0316 (2)	−0.00134 (17)	0.01218 (17)	−0.00316 (18)
Br1	0.0702 (4)	0.0754 (4)	0.0621 (4)	0.0065 (3)	0.0168 (3)	0.0066 (3)
N1	0.0239 (15)	0.0223 (15)	0.0314 (17)	0.0007 (12)	0.0104 (13)	−0.0021 (13)
N2	0.0263 (15)	0.0239 (15)	0.0305 (16)	−0.0017 (12)	0.0136 (13)	0.0001 (13)
N3	0.0244 (15)	0.0224 (15)	0.0341 (17)	−0.0033 (12)	0.0128 (13)	−0.0002 (13)
N4	0.0275 (16)	0.0212 (15)	0.0307 (16)	−0.0026 (12)	0.0141 (13)	−0.0024 (13)
N5	0.0256 (15)	0.0246 (15)	0.0293 (17)	0.0032 (13)	0.0079 (13)	−0.0016 (13)
N6	0.0220 (14)	0.0210 (15)	0.0287 (16)	0.0013 (11)	0.0094 (13)	−0.0008 (12)
N7	0.0227 (15)	0.0253 (15)	0.0293 (16)	0.0009 (12)	0.0111 (13)	−0.0002 (13)
N8	0.0237 (15)	0.0221 (14)	0.0309 (17)	−0.0018 (12)	0.0111 (13)	−0.0019 (13)
C1	0.0236 (17)	0.0230 (17)	0.0250 (18)	0.0022 (14)	0.0089 (15)	0.0004 (15)
C2	0.0193 (17)	0.0291 (18)	0.0270 (19)	0.0036 (14)	0.0083 (15)	0.0006 (15)
C3	0.035 (2)	0.028 (2)	0.043 (2)	0.0000 (17)	0.0139 (19)	−0.0059 (18)
C4	0.033 (2)	0.042 (2)	0.048 (3)	0.0118 (18)	0.022 (2)	−0.001 (2)
C5	0.028 (2)	0.048 (2)	0.039 (2)	0.0058 (18)	0.0154 (18)	0.0071 (19)
C6	0.0262 (19)	0.035 (2)	0.034 (2)	−0.0013 (16)	0.0144 (17)	0.0067 (17)
C7	0.0234 (18)	0.0280 (19)	0.0252 (19)	0.0026 (15)	0.0088 (15)	0.0022 (15)
C8	0.0224 (17)	0.0276 (18)	0.0277 (18)	−0.0003 (15)	0.0104 (15)	0.0063 (16)
C9	0.0239 (18)	0.0228 (17)	0.0289 (19)	−0.0020 (14)	0.0082 (16)	0.0025 (15)
C10	0.0273 (19)	0.0231 (18)	0.030 (2)	−0.0029 (15)	0.0061 (16)	0.0034 (15)
C11	0.035 (2)	0.030 (2)	0.036 (2)	−0.0078 (17)	0.0096 (18)	0.0049 (17)
C12	0.037 (2)	0.035 (2)	0.052 (3)	−0.0131 (18)	0.007 (2)	0.002 (2)
C13	0.053 (3)	0.030 (2)	0.049 (3)	−0.012 (2)	0.015 (2)	−0.011 (2)
C14	0.041 (2)	0.0218 (19)	0.044 (2)	−0.0011 (17)	0.0099 (19)	−0.0040 (17)
C15	0.0302 (19)	0.0231 (18)	0.0266 (19)	−0.0028 (15)	0.0063 (16)	0.0011 (15)
C16	0.0285 (19)	0.0202 (17)	0.0297 (19)	0.0030 (15)	0.0074 (16)	0.0030 (15)
C17	0.0262 (18)	0.0240 (17)	0.0233 (18)	0.0032 (15)	0.0051 (15)	−0.0005 (15)
C18	0.0252 (19)	0.0313 (19)	0.0268 (19)	0.0051 (15)	0.0049 (16)	−0.0023 (16)
C19	0.036 (2)	0.034 (2)	0.041 (2)	0.0067 (17)	0.0140 (19)	−0.0100 (18)
C20	0.046 (3)	0.043 (2)	0.053 (3)	0.009 (2)	0.025 (2)	−0.014 (2)
C21	0.034 (2)	0.050 (3)	0.041 (2)	0.0038 (19)	0.019 (2)	−0.002 (2)
C22	0.0306 (19)	0.035 (2)	0.034 (2)	0.0060 (16)	0.0157 (17)	0.0019 (17)
C23	0.0282 (18)	0.0276 (18)	0.0288 (19)	0.0018 (16)	0.0102 (16)	0.0024 (16)
C24	0.0234 (17)	0.0253 (18)	0.0240 (18)	0.0027 (14)	0.0101 (15)	0.0008 (14)
C25	0.0205 (17)	0.0227 (17)	0.0287 (19)	−0.0007 (14)	0.0102 (15)	0.0011 (15)
C26	0.0233 (17)	0.0208 (17)	0.0295 (19)	−0.0021 (14)	0.0062 (15)	0.0028 (15)
C27	0.0269 (19)	0.032 (2)	0.039 (2)	−0.0040 (16)	0.0133 (17)	0.0049 (17)
C28	0.033 (2)	0.029 (2)	0.049 (3)	−0.0078 (17)	0.0103 (19)	0.0070 (19)
C29	0.038 (2)	0.0238 (19)	0.047 (2)	−0.0064 (17)	0.009 (2)	−0.0039 (18)
C30	0.035 (2)	0.0235 (19)	0.042 (2)	0.0027 (16)	0.0131 (18)	−0.0008 (17)
C31	0.0243 (18)	0.0190 (17)	0.0286 (19)	−0.0013 (14)	0.0062 (15)	0.0015 (14)
C32	0.0195 (16)	0.0198 (16)	0.0267 (18)	0.0012 (13)	0.0071 (15)	−0.0004 (14)
N9	0.043 (2)	0.043 (2)	0.041 (2)	0.0002 (17)	0.0150 (17)	0.0038 (17)
C34	0.070 (3)	0.060 (3)	0.059 (3)	0.004 (3)	0.038 (3)	−0.002 (3)
C35	0.047 (3)	0.066 (3)	0.059 (3)	0.008 (2)	0.025 (2)	0.013 (3)
C36	0.034 (2)	0.072 (3)	0.044 (3)	−0.006 (2)	0.005 (2)	0.014 (2)
N10	0.046 (2)	0.050 (2)	0.037 (2)	−0.0004 (18)	0.0054 (17)	0.0001 (17)
C37	0.036 (2)	0.045 (2)	0.031 (2)	0.0023 (19)	0.0059 (18)	0.0049 (19)
C38	0.055 (3)	0.070 (3)	0.044 (3)	0.015 (2)	0.021 (2)	0.007 (2)
C39	0.045 (3)	0.054 (3)	0.067 (3)	0.007 (2)	0.026 (3)	0.019 (2)
C40	0.042 (3)	0.057 (3)	0.078 (4)	−0.003 (2)	0.011 (3)	0.021 (3)
C41	0.053 (3)	0.050 (3)	0.070 (4)	−0.014 (2)	−0.005 (3)	0.002 (3)
C42	0.056 (3)	0.039 (2)	0.061 (3)	−0.009 (2)	0.015 (2)	0.006 (2)

### Geometric parameters (Å, º)

**Table:**

Zn1—N2	2.032 (3)	C19—H19	0.9300
Zn1—N8	2.032 (3)	C20—C21	1.383 (6)
Zn1—N6	2.034 (3)	C20—H20	0.9300
Zn1—N4	2.040 (3)	C21—C22	1.396 (5)
Zn1—Br1	2.4591 (7)	C21—H21	0.9300
N1—C32	1.327 (4)	C22—C23	1.370 (5)
N1—C1	1.328 (4)	C22—H22	0.9300
N2—C8	1.366 (4)	C23—C24	1.466 (5)
N2—C1	1.370 (4)	C25—C26	1.461 (5)
N3—C8	1.332 (4)	C26—C31	1.388 (5)
N3—C9	1.333 (4)	C26—C27	1.398 (5)
N4—C16	1.365 (4)	C27—C28	1.388 (5)
N4—C9	1.374 (4)	C27—H27	0.9300
N5—C16	1.330 (4)	C28—C29	1.378 (5)
N5—C17	1.332 (4)	C28—H28	0.9300
N6—C24	1.353 (4)	C29—C30	1.371 (5)
N6—C17	1.370 (4)	C29—H29	0.9300
N7—C24	1.336 (4)	C30—C31	1.390 (5)
N7—C25	1.341 (4)	C30—H30	0.9300
N8—C32	1.365 (4)	C31—C32	1.475 (4)
N8—C25	1.365 (4)	N9—C37	1.304 (5)
C1—C2	1.465 (4)	N9—C42	1.479 (5)
C2—C3	1.389 (5)	N9—C34	1.492 (5)
C2—C7	1.405 (5)	C34—C35	1.488 (6)
C3—C4	1.389 (5)	C34—H34A	0.9700
C3—H3	0.9300	C34—H34B	0.9700
C4—C5	1.390 (6)	C35—C36	1.491 (6)
C4—H4	0.9300	C35—H35A	0.9700
C5—C6	1.383 (5)	C35—H35B	0.9700
C5—H5	0.9300	C36—N10	1.428 (5)
C6—C7	1.397 (5)	C36—H36A	0.9700
C6—H6	0.9300	C36—H36B	0.9700
C7—C8	1.442 (5)	N10—C37	1.314 (5)
C9—C10	1.455 (5)	N10—H10	0.87 (2)
C10—C11	1.393 (5)	C37—C38	1.495 (6)
C10—C15	1.398 (5)	C38—C39	1.551 (6)
C11—C12	1.387 (5)	C38—H38A	0.9700
C11—H11	0.9300	C38—H38B	0.9700
C12—C13	1.396 (6)	C39—C40	1.496 (7)
C12—H12	0.9300	C39—H39A	0.9700
C13—C14	1.388 (5)	C39—H39B	0.9700
C13—H13	0.9300	C40—C41	1.483 (7)
C14—C15	1.400 (5)	C40—H40A	0.9700
C14—H14	0.9300	C40—H40B	0.9700
C15—C16	1.467 (5)	C41—C42	1.544 (6)
C17—C18	1.454 (5)	C41—H41A	0.9700
C18—C19	1.379 (5)	C41—H41B	0.9700
C18—C23	1.426 (5)	C42—H42A	0.9700
C19—C20	1.393 (5)	C42—H42B	0.9700
			
N2—Zn1—N8	86.86 (11)	C23—C22—H22	121.1
N2—Zn1—N6	151.95 (12)	C21—C22—H22	121.1
N8—Zn1—N6	86.90 (11)	C22—C23—C18	121.5 (3)
N2—Zn1—N4	86.61 (11)	C22—C23—C24	133.2 (3)
N8—Zn1—N4	152.57 (12)	C18—C23—C24	105.3 (3)
N6—Zn1—N4	86.44 (11)	N7—C24—N6	128.2 (3)
N2—Zn1—Br1	105.68 (8)	N7—C24—C23	121.5 (3)
N8—Zn1—Br1	103.56 (8)	N6—C24—C23	110.3 (3)
N6—Zn1—Br1	102.38 (8)	N7—C25—N8	127.6 (3)
N4—Zn1—Br1	103.85 (8)	N7—C25—C26	123.3 (3)
C32—N1—C1	123.2 (3)	N8—C25—C26	109.0 (3)
C8—N2—C1	108.8 (3)	C31—C26—C27	120.7 (3)
C8—N2—Zn1	124.7 (2)	C31—C26—C25	106.9 (3)
C1—N2—Zn1	124.0 (2)	C27—C26—C25	132.3 (3)
C8—N3—C9	124.1 (3)	C28—C27—C26	116.9 (3)
C16—N4—C9	108.8 (3)	C28—C27—H27	121.5
C16—N4—Zn1	124.0 (2)	C26—C27—H27	121.5
C9—N4—Zn1	124.0 (2)	C29—C28—C27	121.9 (3)
C16—N5—C17	123.4 (3)	C29—C28—H28	119.0
C24—N6—C17	108.5 (3)	C27—C28—H28	119.0
C24—N6—Zn1	124.4 (2)	C30—C29—C28	121.3 (4)
C17—N6—Zn1	124.7 (2)	C30—C29—H29	119.3
C24—N7—C25	123.0 (3)	C28—C29—H29	119.3
C32—N8—C25	109.0 (3)	C29—C30—C31	117.7 (4)
C32—N8—Zn1	124.8 (2)	C29—C30—H30	121.1
C25—N8—Zn1	124.6 (2)	C31—C30—H30	121.1
N1—C1—N2	128.1 (3)	C26—C31—C30	121.3 (3)
N1—C1—C2	122.8 (3)	C26—C31—C32	106.1 (3)
N2—C1—C2	109.1 (3)	C30—C31—C32	132.5 (3)
C3—C2—C7	121.4 (3)	N1—C32—N8	127.9 (3)
C3—C2—C1	133.0 (3)	N1—C32—C31	123.3 (3)
C7—C2—C1	105.6 (3)	N8—C32—C31	108.9 (3)
C2—C3—C4	117.8 (3)	C37—N9—C42	123.1 (4)
C2—C3—H3	121.1	C37—N9—C34	120.7 (4)
C4—C3—H3	121.1	C42—N9—C34	115.9 (4)
C3—C4—C5	120.9 (3)	C35—C34—N9	110.6 (4)
C3—C4—H4	119.6	C35—C34—H34A	109.5
C5—C4—H4	119.6	N9—C34—H34A	109.5
C6—C5—C4	121.7 (3)	C35—C34—H34B	109.5
C6—C5—H5	119.1	N9—C34—H34B	109.5
C4—C5—H5	119.1	H34A—C34—H34B	108.1
C5—C6—C7	117.9 (3)	C34—C35—C36	109.6 (4)
C5—C6—H6	121.0	C34—C35—H35A	109.8
C7—C6—H6	121.0	C36—C35—H35A	109.8
C6—C7—C2	120.2 (3)	C34—C35—H35B	109.8
C6—C7—C8	132.7 (3)	C36—C35—H35B	109.8
C2—C7—C8	107.2 (3)	H35A—C35—H35B	108.2
N3—C8—N2	127.3 (3)	N10—C36—C35	110.4 (4)
N3—C8—C7	123.4 (3)	N10—C36—H36A	109.6
N2—C8—C7	109.3 (3)	C35—C36—H36A	109.6
N3—C9—N4	127.1 (3)	N10—C36—H36B	109.6
N3—C9—C10	123.6 (3)	C35—C36—H36B	109.6
N4—C9—C10	109.3 (3)	H36A—C36—H36B	108.1
C11—C10—C15	120.8 (3)	C37—N10—C36	124.3 (4)
C11—C10—C9	132.6 (3)	C37—N10—H10	118 (1)
C15—C10—C9	106.5 (3)	C36—N10—H10	118 (1)
C12—C11—C10	117.3 (4)	N9—C37—N10	121.2 (4)
C12—C11—H11	121.4	N9—C37—C38	121.0 (4)
C10—C11—H11	121.4	N10—C37—C38	117.9 (4)
C11—C12—C13	121.9 (4)	C37—C38—C39	114.3 (4)
C11—C12—H12	119.0	C37—C38—H38A	108.7
C13—C12—H12	119.0	C39—C38—H38A	108.7
C14—C13—C12	121.3 (4)	C37—C38—H38B	108.7
C14—C13—H13	119.4	C39—C38—H38B	108.7
C12—C13—H13	119.4	H38A—C38—H38B	107.6
C13—C14—C15	116.8 (4)	C40—C39—C38	114.9 (4)
C13—C14—H14	121.6	C40—C39—H39A	108.5
C15—C14—H14	121.6	C38—C39—H39A	108.5
C10—C15—C14	121.8 (3)	C40—C39—H39B	108.5
C10—C15—C16	106.5 (3)	C38—C39—H39B	108.5
C14—C15—C16	131.6 (3)	H39A—C39—H39B	107.5
N5—C16—N4	127.9 (3)	C41—C40—C39	116.5 (4)
N5—C16—C15	123.2 (3)	C41—C40—H40A	108.2
N4—C16—C15	109.0 (3)	C39—C40—H40A	108.2
N5—C17—N6	127.4 (3)	C41—C40—H40B	108.2
N5—C17—C18	122.5 (3)	C39—C40—H40B	108.2
N6—C17—C18	110.0 (3)	H40A—C40—H40B	107.3
C19—C18—C23	120.4 (3)	C40—C41—C42	114.4 (5)
C19—C18—C17	133.7 (3)	C40—C41—H41A	108.7
C23—C18—C17	105.9 (3)	C42—C41—H41A	108.7
C18—C19—C20	117.2 (4)	C40—C41—H41B	108.7
C18—C19—H19	121.4	C42—C41—H41B	108.7
C20—C19—H19	121.4	H41A—C41—H41B	107.6
C21—C20—C19	122.5 (4)	N9—C42—C41	112.9 (3)
C21—C20—H20	118.8	N9—C42—H42A	109.0
C19—C20—H20	118.8	C41—C42—H42A	109.0
C20—C21—C22	120.6 (4)	N9—C42—H42B	109.0
C20—C21—H21	119.7	C41—C42—H42B	109.0
C22—C21—H21	119.7	H42A—C42—H42B	107.8
C23—C22—C21	117.7 (4)		

### Hydrogen-bond geometry (Å, º)

**Table:**

*D*—H···*A*	*D*—H	H···*A*	*D*···*A*	*D*—H···*A*
N10—H10···Br1	0.87 (2)	2.44 (2)	3.281 (4)	163 (2)

### Table S1 Comparison of Zn—L bond length and displacement of Zn from N_4_ plane (Zn—N_4_) in 1 and different literature ZnPc complexes (Å)

**Table:**

Compound	Zn—L	Zn—N_4_	Ref.
ZnPc—(DBU)	2.063 (2)	0.614 (2)	Janczak *et al.*, 2011
ZnPc—(4-picoline)	2.166 (3)	0.352 (2)	Kubiak *et al.*, 2007
ZnPc—(2-amino-3-picoline)	2.157 (2)	0.470 (2)	Janczak *et al.*, 2009
ZnPc—(n-buthylamine)	2.073 (4)	0.494 (4)	Przybył & Janczak, 2014
ZnPc—(n-amylamine)	2.087 (3)	0.498 (4)	Przybył & Janczak, 2014
ZnPc—(n-heptylamine)	2.097 (2)	0.477 (4)	Przybył & Janczak, 2014
ZnPc—(n-hexylamine)	2.178	0.480	Kobayashi *et al.*, 1971
ZnPc—(pyrazine)	2.178 (2)	0.371 (2)	Janczak & Kubiak, 2009
(ZnPc)_2_—(pyrazine)	2.207 (2)	0.296 (2)	Janczak & Kubiak, 2009
ZnPc—(4-aminopyridine)	2.092 (3)	0.446 (1)	Yang *et al.*, 2008
ZnPc—(1,3-bis(4-pyridyl)propane)	2.130 (5)	0.375 (3)	Zeng *et al.*, 2005
(ZnPc)_2_—(µ_2_-1,2-bis(4-pyridyl)ethane)	2.125 (7)	0.483 (4)	Zeng *et al.*, 2005
(ZnPc)_2_—(µ_2_-1,2-bis(4-pyridyl)ethylene)	2.156 (2)	0.347 (2)	Zeng *et al.*, 2005
(ZnPc)_2_—(µ_2_-N,N'-bis((pyridin-3-yl)methyl)ethanediamide)	2.147 (3)	0.377 (3)	Li *et al.*, 2011
ZnPc—Cl	2.35	0.59	Mossoyan-Deneux *et al.*, 1985
ZnPc—Br	2.459 (3)	0.488 (3)	this work
